# Long‐Distance, Transfrontier Carnivore Dispersals in Southern Africa

**DOI:** 10.1002/ece3.70574

**Published:** 2024-11-11

**Authors:** Piet Beytell, Lise Hanssen, Ortwin Aschenborn, Robin Naidoo

**Affiliations:** ^1^ Ministry of Environment Forestry & Tourism Windhoek Namibia; ^2^ Kwando Carnivore Project Kongola Namibia; ^3^ Leibnitz Institute for Zoo and Wildlife Research Windhoek Namibia; ^4^ WWF‐US Washington DC USA; ^5^ University of British Columbia Vancouver British Columbia Canada

**Keywords:** carnivores, dispersal, hyperdispersal, natal dispersal, Southern Africa, transfrontier

## Abstract

Information on long‐range dispersal in tropical carnivores is limited compared to their temperate counterparts. Here we present data on long‐range, transboundary dispersals for three species of tropical carnivores: African wild dog ( *Lycaon pictus* ), African lion ( *Panthera leo* ), and spotted hyena ( *Crocuta crocuta* ). The dispersals we document in our savannah system are among the longest that have been recorded for African wild dog and African lion, while for spotted hyena one of our recorded dispersal events is of similar magnitude to the longest documented movements occurring among any carnivore species from around the world.

## Graphical abstract

Information on long‐range dispersal in tropical carnivores is limited compared to their temperate counterparts. We document tropical carnivore dispersals that are among the longest that have been recorded for African wild dog and African lion, while for spotted hyena one of our recorded dispersal events is on par with the longest documented movements occurring among any carnivore species from around the world.

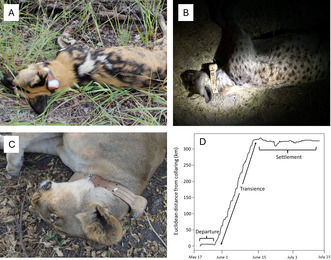

## 1 Introduction

Long‐distance dispersal in carnivores has been extensively documented in North America and Europe (e.g., Packila et al. 2017; Bartoń et al. 2019), but there is less information on such carnivore movements in other parts of the world. GPS tracking has revolutionized our understanding of animal movements across the globe, but as with other types of ecological studies (Martin, Blossey, and Ellis 2012), movement ecology research remains heavily biased toward sites in the northern hemisphere (Supp et al. 2021). Indeed, there is little information on the movement of several large, southern hemisphere carnivores (e.g., jaguar *Panthera onca* , snow leopard *Panthera uncia* , clouded leopard *Neofelis nebulosa* ) during the dispersal process (de Oliveira et al. 2022). Here, we add to the literature on tropical carnivore dispersal in savannah systems and report on long‐distance dispersal events for African wild dog ( *Lycaon pictus* ), African lion ( *Panthera leo* ), and spotted hyena ( *Crocuta crocuta* ) that are among the longest recorded for each species. Our dataset on the movement of 191 individuals across three countries in southern Africa includes a spotted hyena dispersal event that is on par with the longest documented dispersal movements for any carnivore species.

Natal dispersal (i.e., the permanent movement of individuals from their birth site to their first breeding or potential breeding range) in carnivore species is common and as with other mammals is typically sex‐biased, with males often dispersing across distances that are many times greater than females (Greenwood 1980; Waser and Jones 1983). Furthermore, “hyperdispersal” (i.e., long‐range dispersal after an animal has been translocated from its native home range) has also been observed among a wide variety of taxa (Bilby and Moseby 2024). Distances of both dispersal types can be as much as an order of magnitude greater than the linear distances traveled within average annual home ranges (de Oliveira et al. 2022), and astounding accounts of long‐range dispersal treks by species such as Canada lynx ( *Lynx canadensis* ), arctic fox ( *Vulpes lagopus* ), and gray wolf ( *Canis lupus* ) have captivated scientific and popular audiences alike (e.g., https://www.denverpost.com/2010/04/16/lynx‐relocated‐to‐colorado‐traveled‐1200‐miles‐back‐to‐canada/). Because it tends to be difficult to locate and tag individuals that are on the verge of dispersing, and because sample sizes in GPS tagging exercises are low relative to other types of ecological efforts, capturing these events is often a haphazard process rather than one of systematic design. As such, the frequency with which such long‐range movements occur is difficult to quantify (Matshisela et al. 2022; Dejid et al. 2022). This effect is even more pronounced in tropical and/or developing countries, where GPS tagging studies are heavily outnumbered by those in temperate and/or developed countries (Kays et al. 2022).

## 2 Materials and Methods

**FIGURE 1:**
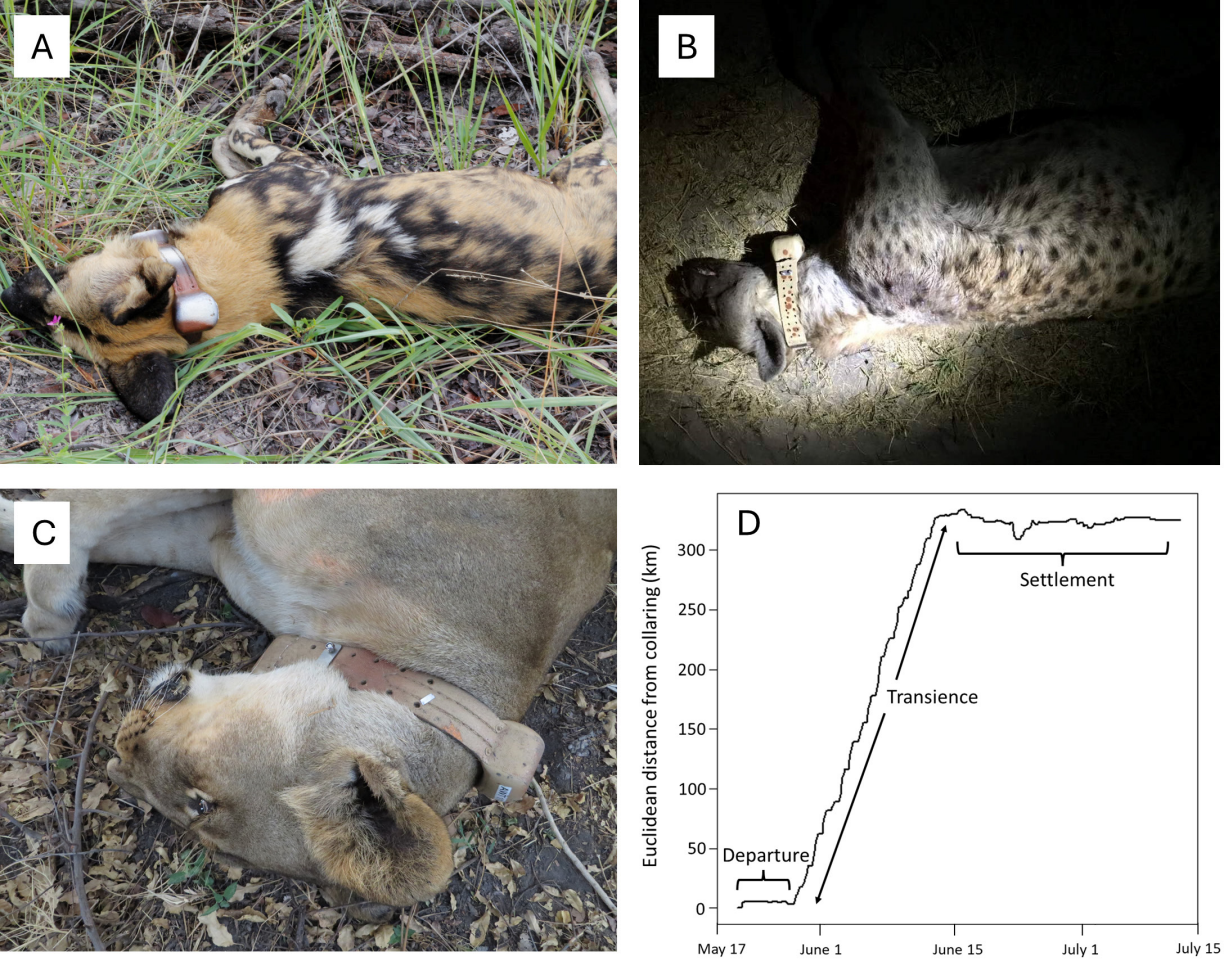
Deploying satellite tracking collars on African wild dog (A), spotted hyena (B), and African lion (C), and Euclidean or straight‐line distance (the square root of Net‐Squared Displacement (NSD)) for a female lion that dispersed from Bwabwata National Park, Namibia, to Makgadikgadi Pans National Park, Botswana, over a 2‐month period after collar deployment on May 22, 2022 (D).

Our applied research program on carnivore movement ecology in Namibia and surrounding southern African countries spans the years 2008 to present and to date has tagged over 200 individuals from six species. We present data from the three most abundant species in our dataset: African wild dog (*n* = 21 individuals), spotted hyena (*n* = 46), and African lion (*n* = 124). GPS collars were deployed in national parks and communal lands in northeast Namibia on sub‐adult and adult individuals (Figure 1), with estimated ages ranging from 2 to 8 years old. Research permits that granted permission to immobilize and tag animals were obtained from Namibia's Ministry of Environment, Forestry and Tourism, the management authority that oversees such efforts. Collars were programmed to record GPS locations at intervals ranging from 1 to 24 h (the most frequent intervals were between 4 and 5 h), with a median collar lifespan of 316 days (range 9–834). To identify dispersing individuals, we used a net‐squared displacement (NSD) approach and visually examined plots of NSD versus time since collaring for all collared individuals (Singh et al. 2012; Spitz, Hebblewhite, and Stephenson 2017). Dispersing individuals were readily characterized by plots (e.g., Figure 1D) that showed an initial stable NSD trajectory over time (departure phase), followed by a subsequent rise (transience phase) and eventual stabilization at a new, higher average NSD (settlement phase). Identifying this characteristic pattern of displacement distance over time for dispersing individuals via visual inspection of NSD plots has been used for various species and contexts (e.g., Mysterud et al. 2011; Killeen et al. 2014).

## 3 Results and Discussion

**FIGURE 2:**
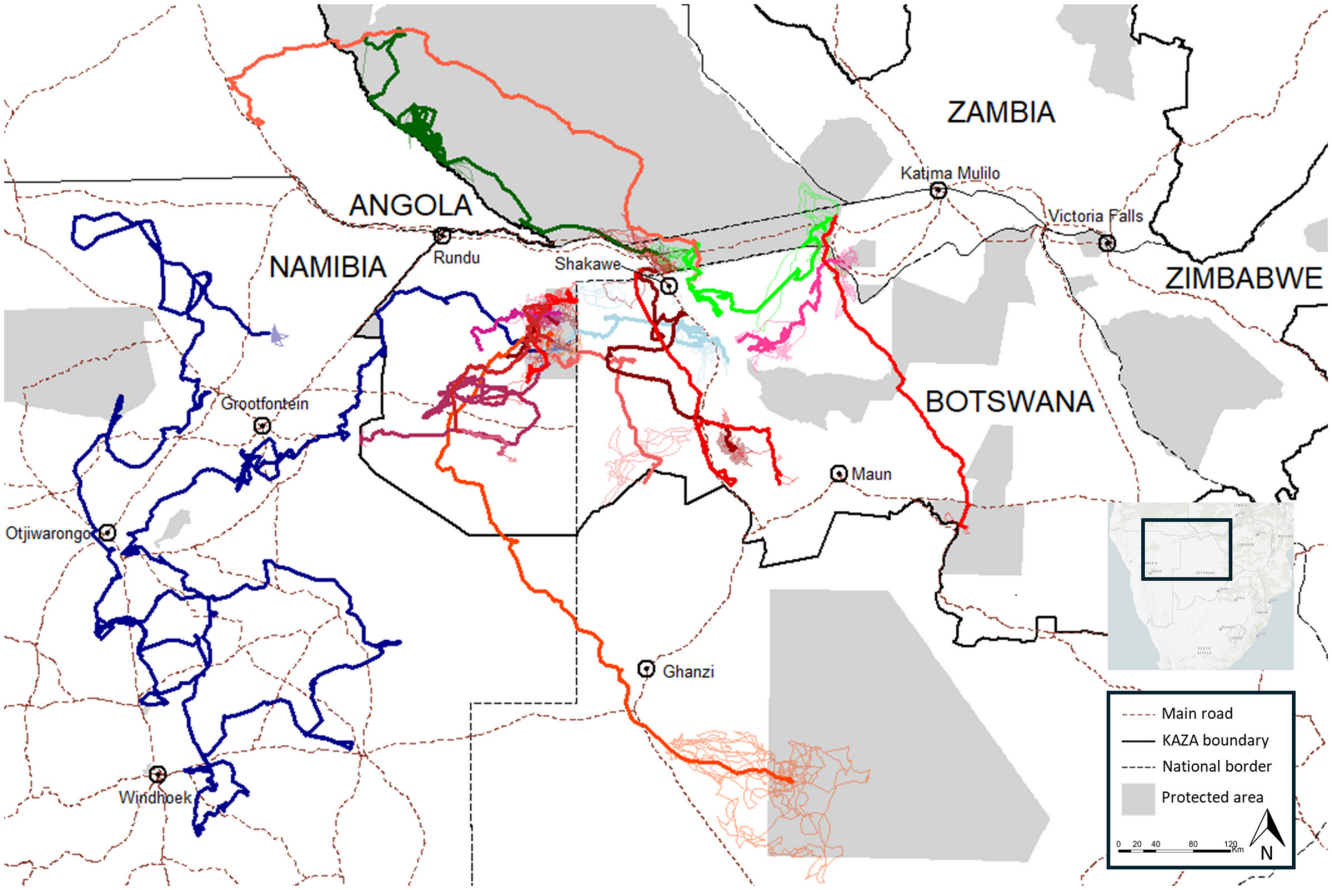
Movement trajectories for dispersing African wild dogs (green shades), spotted hyenas (blue shades), and African lions (red shades) in the Kavango‐Zambezi transfrontier conservation area (KAZA). Heavier lines indicate the “transience” phase of dispersal, characterized by directed, long‐distance movements.

We identified two African wild dogs (9.5% of the individuals collared for this species), 13 lions (10.5%), and two spotted hyenas (4.3%) that dispersed during the time period in which they were collared (Figure 2). Of the wild dog dispersers, an adult female (Table 1, individual “WD1”) that was collared at the eastern end of Bwabwata National Park moved through northern Botswana and settled 147 km away on the western side of Bwabwata. The other individual, an adult male (Table 1, “WD2”), eventually settled 244 km from where it was collared in Bwabwata and covered an overall distance of at least 3144 km (with a maximum straight‐line distance from its collaring location of 342 km) during its travels, most of which were in southern Angola's Luengue‐Luiana National Park. These latter two measurements are slightly lower than the longest African wild dog dispersal distances documented to date, although longer than most other notable long‐distance dispersals that have been documented for this species (summarized by Sandoval‐Seres et al. 2022). Dispersals documented via GPS data in the Serengeti ecosystem in East Africa (straight‐line distance of 520, 3892 km covered; Masenga et al. 2015) and observations of two long‐distance dispersals via identification of known individuals in Zimbabwe (inferred straight‐line distances of 476 and 447 km) exceeded the straight‐line dispersal distances of African wild dog recorded in our study.

**TABLE 1:** Transience phase movement parameters for 17 dispersing carnivores.

Species	ID	Sex	Trans‐located?	Duration			Distance (km)			Directedness	
				Initiation	Termination	Days	Average daily displacement^a^	Straight‐line^b^	Total^c^	Average daily turning angle^d^	Straightness^e^
African wild dog	WD1	Male	*n*	2014‐04‐12	2014‐05‐19	37	9.5	196	514	0.083	0.35
African wild dog	WD2	Male	*n*	2016‐04‐22	2016‐08‐23	123	9.3	342	1683	0.026	0.29
Spotted hyena	SH1	Male	*n*	2019‐02‐05	2019‐02‐27	22	11.9	78	506	0.16	0.55
Spotted hyena	SH2	Male	*n*	2021‐11‐30	2022‐08‐11	254	14.2	390	5165	0.093	0.08
African lion	AL1	Male	*n*	2014‐04‐10	2014‐07‐03	84	8.2	216	818	0.054	0.65
African lion	AL2	Male	*n*	2016‐03‐12	2016‐03‐17	5	7.9	83	83	0.0007	0.79
African lion	AL3	Male	*n*	2013‐05‐10	2013‐06‐09	30	8.0	208	329	0.19	0.61
African lion	AL4	Male	*n*	2018‐04‐03	2018‐05‐14	41	5.7	67	338	0.0001	0.44
African lion	AL5	Male	*y*	2015‐11‐30	2016‐03‐25	116	7.9	252	1181	0.13	0.2
African lion	AL6	Male	*n*	2019‐06‐28	2019‐09‐09	73	6.0	465	777	0.16	0.18
African lion	AL7	Male	*n*	2020‐03‐30	2020‐04‐30	31	3.5	107	188	0.35	0.65
African lion	AL8	Male	*n*	2020‐05‐12	2020‐06‐04	23	15.2	121	485	0.08	0.47
African lion	AL9	Male	*n*	2022‐03‐19	2022‐05‐08	50	5.2	58	351	0.01	0.54
African lion	AL10	Male	*n*	2023‐03‐22	2023‐04‐23	32	11.9	104	485	0.2	0.56
African lion	AL11	Male	*y*	2022‐03‐11	2022‐04‐27	47	4.7	61	349	0.13	0.49
African lion	AL12	Male	*y*	2022‐08‐19	2022‐10‐26	68	9.3	599	786	0.03	0.64
African lion	AL13	Female	*y*	2023‐05‐28	2023‐06‐12	15	17.8	166	383	0.09	0.94

^a^ Average straight‐line distance between fixes at 24‐h intervals during transience phase.

^b^ Straight‐line distance from start to end of transience phase.

^c^ Sum of movement steps from the transience phase.

^d^ In radians; values range from 0 (straight line) to 3.14 (180° movement).

^e^ Straight‐line distance divided by total distance covered during transience phase.

Of the 13 dispersing lions, of which four exhibited post‐translocation hyperdispersal (the only four hyperdispersal events in our dataset), three dispersal trajectories stand out as among the longest‐recorded such events. An adult female lion (Table 1, “AL13”) translocated into eastern Bwabwata National Park subsequently traveled south in a highly directional manner through northern Botswana into the Makgadikgadi Pans National Park, covering at least 383 km and ended up 327 km in a straight line away from her point of collaring. A second, adult male lion (Table 1, “AL12”) translocated into Bwabwata National Park traveled at least 786 km through Luengue‐Luiana National Park, ultimately crossing the Kavango River into unprotected lands in southern Angola, at which point the animal was killed at a straight‐line distance from point of collaring of 443 km. Finally, a third, 4‐year‐old male lion (Table 1, “AL1”) collared in Khaudum National Park dispersed and traveled 818 km to the Central Kalahari Game Reserve in Botswana (a straight‐line distance of 470 km during the transience phase, with a maximum straight‐line distance after settlement of 574 km) where he established a large, 15,840 km^2^ home range for the next 3 months. The three long‐distance movements that we document here are among the longest lion dispersals ever recorded, with previous research documenting long‐distance dispersal movements of 105 km in the Sebungwe region of Zimbabwe (Matshisela et al. 2022), ~200 km in Kruger National Park in South Africa (van Hooft et al. 2018), ~250 km from Chobe National Park to the Central Kalahari Game Reserve (Finerty et al. 2023) in Botswana, and a maximum implied distance from genetic analyses of 471 km in the Hwange‐Chobe region of Zimbabwe/Botswana (Dures et al. 2021).

Perhaps most notably, one of the two dispersing spotted hyenas, a young male collared in Khaudum National Park in 2021 traveled a cumulative distance of at least 6463 km (Table 1, “SH2”), with a transience phase lasting nearly 9 months, across a large swathe of northern and central Namibia (Figure 2, heavy dark blue line). At its furthest, the animal was 589 km in a straight line from the point where it was collared, after which it continued its dispersal and eventually settled in communal lands 277 km from its initial recorded location. The astounding distance that was covered by this animal is on par with other long‐distance movement records—typically made during dispersal—of northern hemisphere carnivores such as arctic fox (Fuglei and Tarroux 2019), Canada lynx (Poole 2003), red fox *Vulpes vulpes* (Walton et al. 2018), gray wolf (Mech 2020), brown bear *Ursus arctos* (Bartoń et al. 2019), and wolverine *Gulo gulo* (Packila et al. 2017). Furthermore, we were unable to find published references to a longer documented dispersal movement for any terrestrial carnivore species in the southern hemisphere.

**FIGURE 3:**
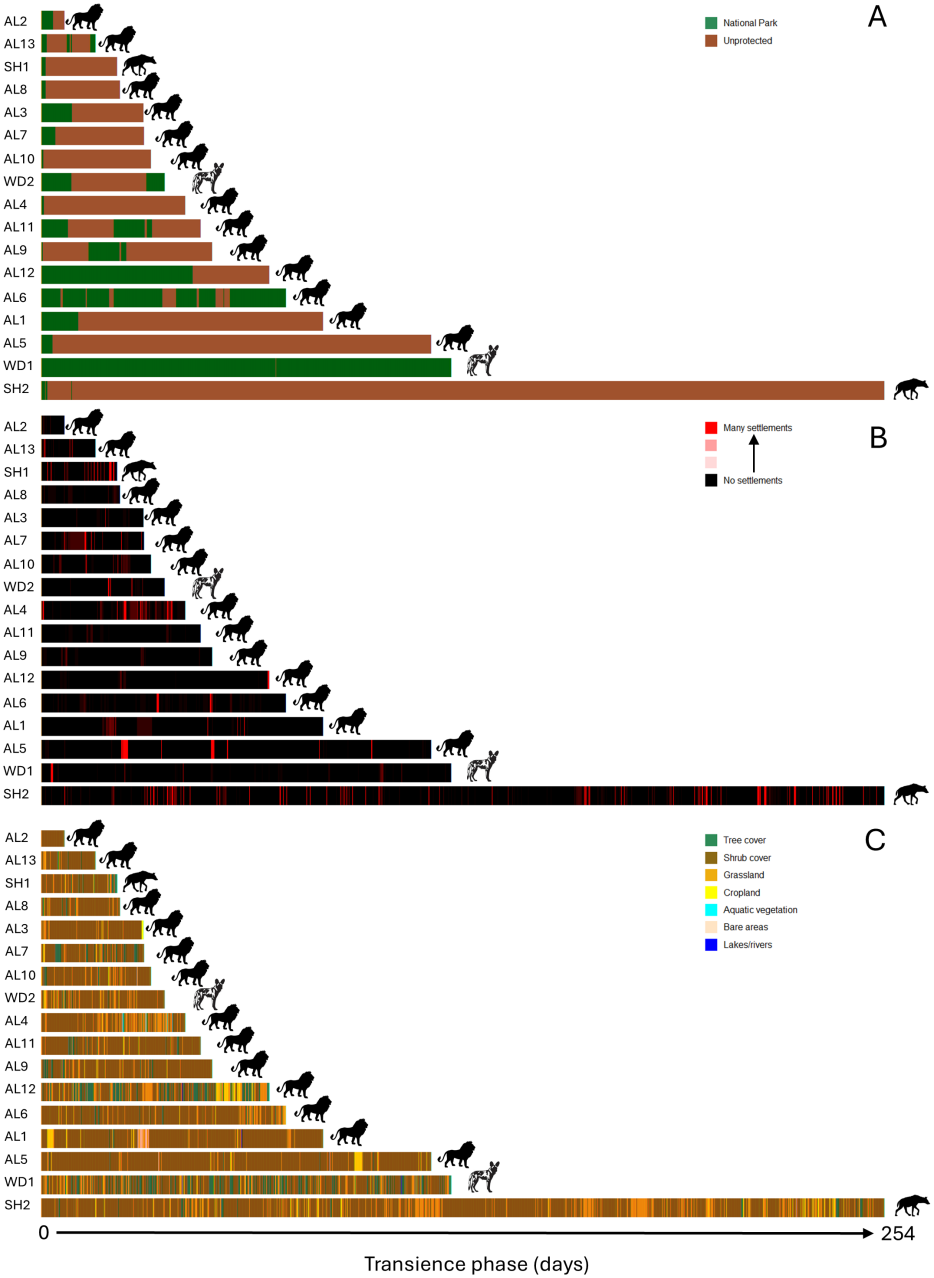
Use of protected or unprotected lands (A), proximity to human settlements (B), and types of land cover used (C) during the transience phase of dispersal for 17 southern African carnivores. Each vertical bar represents one (3‐h interval) GPS observation.

Beyond the sheer distance covered and the extended duration, additional aspects of the transience phase of this far‐ranging male hyena SH2 differed compared to the other 16 long‐range dispersers. Of all dispersing carnivore individuals, SH2 had the least straight (straightness is the ratio of straight‐line distance between start and end point to total path length; Benhamou 2004) movement trajectory during the transience phase (Table 1, column 12). Furthermore, the hyena at one stage was within 2.5 km of Namibia's international airport near the capital city of Windhoek, and other than a 3‐day visit to Etosha National Park, spent its entire transience phase outside of protected areas after leaving Khaudum National Park (Figure 3A). On the contrary, the density of settlements (Marconcini et al. 2020) within a 1‐km radius of GPS fixes and the land cover types (https://www.esa.int/ESA_Multimedia/Images/2017/10/African_land_cover) SH2 used were within the range of values for other long‐range dispersers during their transience phase (Figure 3B,C). While his motivations for dispersing as long, as far, and in as tortuous a manner as he did remain a mystery, the epic journey of this young male hyena through large areas of private and communal farmland, where landholders tend to be hostile to large predators (Schumann, Walls, and Harley 2012), is a testament to the remarkable potential of carnivores to travel discreetly through anthropogenic landscapes (Cushman et al. 2016).

Although the dispersal distance and duration of spotted hyena SH2 stand out among its collared peers, there was a high degree of variability in movement metrics across all dispersing individuals. Focusing just on the transience phase of dispersal, the length of this period varied from 5 to 283 days (mean = 62); that is, from less than a week to over 9 months (Table 1, column 7). Total distance covered (Table 1, column 10), straight‐line displacement (Table 1, column 9), and average daily displacement (Table 1, column 8) each varied over an order of magnitude, and the straightness of dispersal transience trajectories varied across nearly the full range of possible values (0–1; Table 1, column 12). Such substantial variation in dispersal attributes from a relatively small set of collared individuals suggests there is still much to learn regarding the dispersal process within these three species, let alone across less well‐studied tropical carnivores.

Nine of the 18 long‐distance dispersers crossed at least one country border, with two of the dispersing African wild dogs and three lions moving through three countries (Angola, Botswana, Namibia). Most, though not all, of the area covered by long‐distance dispersers of these three species, falls within the Kavango‐Zambezi transfrontier conservation area (“KAZA”), a five‐country initiative where one of the goals is to better manage cross‐border animal movements, including those of predators (KAZA Carnivore Conservation Coalition 2018). Nevertheless, five of the 13 dispersing lions were killed by people during the lifetime of their collars, including three of the five animals that dispersed out of Namibia and into either the Angolan or Botswanan part of KAZA. While connectivity research often focuses on the identification of movement corridors (e.g., Blazquez‐Cabrera et al. 2019), in the case of wide‐ranging, dispersing carnivores a more appropriate strategy to improve connectivity and promote population persistence or expansion may be to improve human tolerance and social permeability for carnivores across entire landscapes (Ghoddousi et al. 2021). Such efforts are already being successfully undertaken in parts of KAZA (Leflore et al. 2020), but more work is needed to secure the future of large carnivores in this globally significant conservation landscape.

## Author Contributions

**Piet Beytell:** conceptualization (equal), data curation (lead), funding acquisition (lead), investigation (lead), methodology (lead), writing – original draft (equal), writing – review and editing (equal). **Lise Hanssen:** conceptualization (equal), data curation (lead), funding acquisition (lead), investigation (lead), methodology (lead), writing – original draft (equal), writing – review and editing (equal). **Ortwin Aschenborn:** data curation (equal), investigation (equal), methodology (equal), writing – original draft (equal), writing – review and editing (equal). **Robin Naidoo:** conceptualization (equal), data curation (equal), formal analysis (lead), visualization (lead), writing – original draft (equal), writing – review and editing (equal).

## Conflicts of Interest

Piet Beytell, Lise Hanssen, and Robin Naidoo work for organizations whose mandate includes support for the Kavango‐Zambezi transfrontier conservation area.

## Data Availability

Data supporting this research are restricted and not available publicly. Detailed movement data on threatened and/or sensitive species such as the three carnivores we discuss are owned by the Ministry of Environment, Forestry, and Tourism, Government of Namibia and are available to qualified researchers only by contacting the Principal Scientist, Directorate of Scientific Service, Ministry of Environment, Forestry, and Tourism, Government of Namibia and requesting the data used in this manuscript (current email: piet.beytell@meft.gov.na).
